# Radiation losses in the microwave K_u_ band in magneto-electric nanocomposites

**DOI:** 10.3762/bjnano.6.173

**Published:** 2015-08-07

**Authors:** Talwinder Kaur, Sachin Kumar, Jyoti Sharma, A K Srivastava

**Affiliations:** 1 Department of Physics, Lovely Professional University, Phagwara-144411, India; 2 Department of Chemistry, Guru Nanak Dev University, Amritsar-143005, India

**Keywords:** emulsion polymerization, magneto-electric composite, radiation loss, vector network analyser

## Abstract

A study on radiation losses in conducting polymer nanocomposites, namely La–Co-substituted barium hexaferrite and polyaniline, is presented. The study was performed by means of a vector network analyser, X-ray diffraction, Fourier transform infrared spectroscopy, transmission electron microscopy, electron spin resonance spectroscopy and a vibrating sample magnetometer. It is found that the maximum loss occurs at 17.9 GHz (−23.10 dB, 99% loss) which is due to the composition of a conducting polymer and a suitable magnetic material. A significant role of polyaniline has been observed in ESR. The influence of the magnetic properties on the radiation losses is explained. Further studies revealed that the prepared material is a nanocomposite. FTIR spectra show the presence of expected chemical structures such as C–H bonds in a ring system at 1512 cm^−1^.

## Introduction

Conducting polymer nanocomposites have been in the focus of research mainly because of a number of attractive technological applications in the microwave region such as radar and radar-absorbing materials [1–2], Wi-Fi systems and other communication systems, and microwave devices. They also have great potential for other applications such as rechargeable batteries. The extensive use of electrical equipment, producing microwave, has created a new problem named electromagnetic interference (EMI), which is not good for device health. So, an effective material is required to reduce the electromagnetic noise by using absorbing materials. Earlier, carbon black, metal flakes, iron balls and recently carbon nanotubes with magnetic particles were used as absorbent. However, there are some limitations such as a fixed absorption range, difficulties during synthesis, impedance matching problems [3].

From theoretical concepts for radiation losses, it is found that magnetic, dielectric and conducting properties directly influence the absorption capacity. So, a composite with magnetic material and a conducting polymer having a conjugated π-system is required to meet these desired characteristics [4]. Polyaniline has caught much attention because of its environmental stability, easy synthesis process and high electrical conductivity. From the magnetic point of view, hexaferrite can play a key role with high saturation magnetization and coercivity. The magnetic loss of hexaferrite and the electric loss of the polymer contribute to high absorption over a wide range [5]. Hence, researchers turned their attention towards magnetic particles embedded in polymers [6–12] with a focus on composites of hexaferrite and conducting polymers [13–18].

M-type hexaferrite is an interesting material with variable properties and a large anisotropy field having a magnetic resonance in the range of 2–52 GHz. Also, at the nano-scale, its optical properties [19], magnetic properties [20–21], piezo-electric properties [22], photocatalytic properties [23], gas-sensing properties [24], electrical properties, dielectric properties [25–26], and mechanical properties [27] are better than those of the bulk material. Hexaferrite is also extensively studied by researchers for radiation absorption which is based on magnetic resonance phenomena because of the anisotropy field [10,28]. In this paper, we have used the versatile citrate precursor method to synthesize La–Co-substituted barium hexaferrite. Lanthanum and cobalt are used as substituents to enhance the magnetic properties of barium hexaferrite and then emulsion polymerization is employed for the synthesis of nanocomposites. The structural, magnetic and radiation absorbance properties of synthesized compounds have been investigated by using X-ray diffraction (XRD), Fourier transform infrared spectroscopy (FTIR), electron spin resonance spectroscopy (ESR), transmission electron microscopy (TEM), vibrating sample magnetometry (VSM) and vector network analyser (VNA).

## Experimental

### Synthesis

The synthesis of La–Co-substituted barium hexaferrite has been carried out through a sol–gel method [19] using metal nitrates (AR grade chemicals) without any purification. Aqueous solutions of iron and metal salts are mixed with each other in stoichiometric proportions, at ambient temperature under constant magnetic stirring. Citric acid acts as fuel and chelating agent and is added to the salt solution in a molar ratio of cations to citric acid of 1:1.5. To obtain the fine particles and to enhance the reaction mechanism, ammonium hydroxide (NH_4_OH) solution is added drop wise to maintain a pH value of 6.8. The solution is heated at 80–85 °C for 4–6 h under continuous stirring during which it turns into a brown gel. Then the hot plate is used to make a precursor at 280–300 °C for 3 h. Pre-sintering has been done at 500 °C for 2 h at a rate of 23 °C/min to remove impurities. Then the precursor material is calcined at 900 °C at a rate of 23 °C/min for 5 h.

An aqueous solution (0.3 M) of dodecyl benzene sulfonic acid (DBSA) is added to a barium hexaferrite solution (0.1 M) to form an emulsion. Aniline (0.1 M) is added to the solution. The magnetic material (BaM) and aniline are mixed in the ratio of 1:1. The solution is stirred 3–4 h at very low temperature for micelle formation. To initiate the polymerization, ammonium persulfate is used as an initiator that initiates the reaction at 2 °C. The obtained product is filtered with a suction pump and rinsed with isopropyl alcohol then with distilled water. Then samples are placed in oven and dried at 85 °C for 8 h [4].

### Characterization

X-ray powder diffraction patterns are obtained with a Bruker AXS D8 Advance X-ray diffractometer in the range of 20–80° using Cu Kα radiation (40 kV and 35 mA, step size 0.02°). Attached functional groups have been analysed with Fourier transform infrared spectrometry (FTIR interferometer IR prestige-21 FTIR (model-8400S)) in the range of 400–4000 cm^−1^ by making calcined product pallets with KBr in a weight ratio of 1:10. ESR measurements were performed at room temperature by using an X-band JEOL JES-ME spectrometer. ESR spectra are recorded under following experimental conditions: magnetic field sweep rate of 50 mT/min, modulation width of 0.35 mT, modulation frequency of 100 kHz, and microwave power of approx. 10 mW (9.5 GHz). Magnetic properties have been studied with a vibrating sample magnetometer (Lakeshore 7410) at room temperature. Microwave studies have been carried out with vector network analyser (Agilent 8722ES) by pressing the powder and making samples of 2 mm thickness (15.8 mm × 7.9 mm). Transmission electron microscopy (TEM) images of samples have been recorded using a JEOL JEM 2100 instrument.

## Results and Discussion

### Phase identification

**Figure 1:**
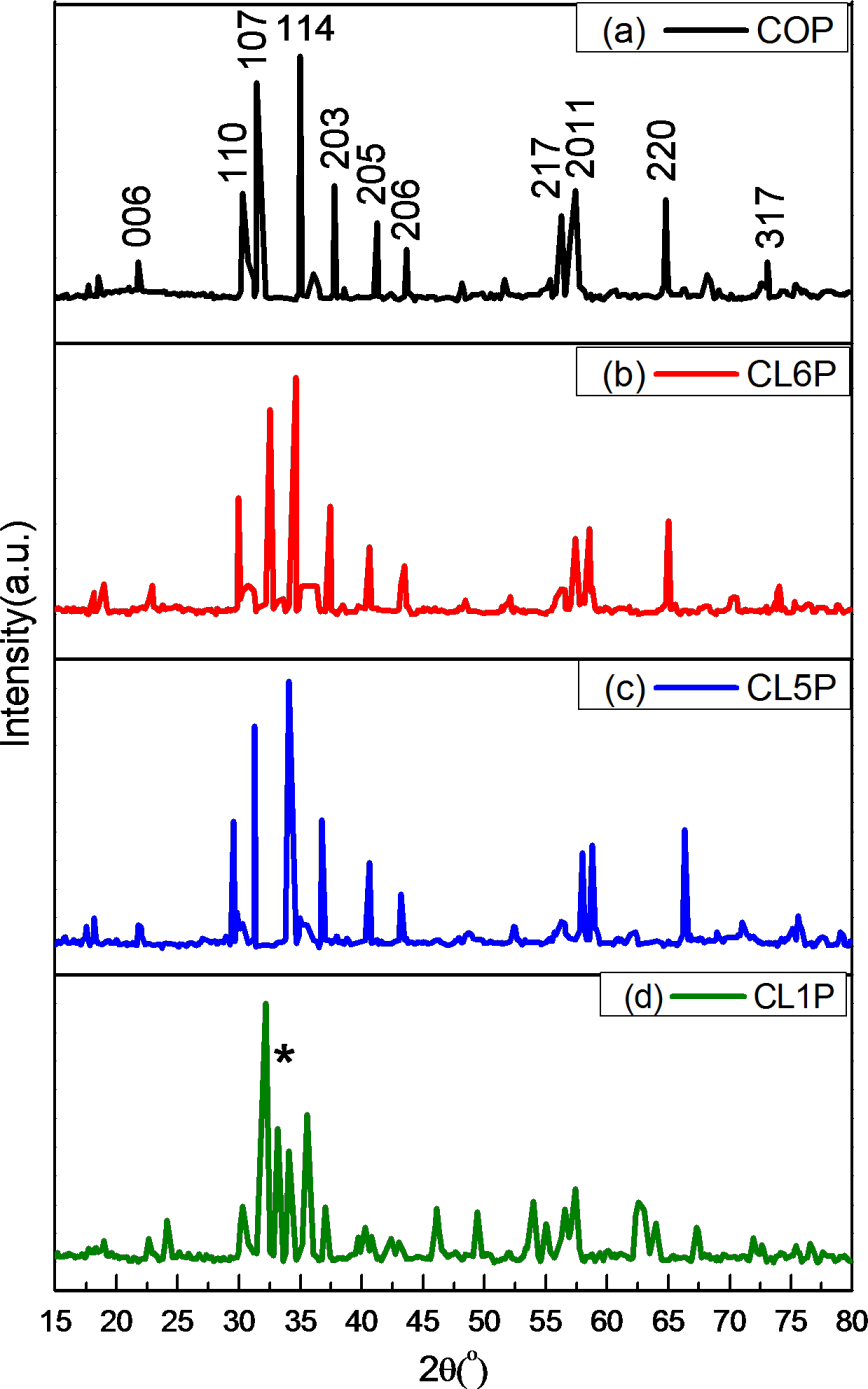
X-ray diffraction pattern of Ba_1−_*_x_*La*_x_*Co*_x_*Fe_12−_*_x_*O_19_/polyaniline composites: (a) *x* = 0.0 (COP), (b) *x* = 0.6 (CL6P), (c) *x* = 0.5 (CL5P) and (d) *x* = 0.1 (CL1P).

The X-ray diffractograms of the hexaferrite Ba_1−_*_x_*La*_x_*Co*_x_*Fe_12−_*_x_*O_19_ (*x* = 0.0, 0.1, 0.5, 0.6)/polyaniline nanocomposites are presented in Figure 1. All samples show a crystalline phase and the absence of impurities (except sample CL1P). The peaks shown in Figure 1 [19] confirm the hexagonal structure of composites and are identical to the peaks in the standard pattern (JCPDS-391433). This proves that the substituted ions have occupied crystal sites. A peak of α-Fe_2_O_3_ appears in Figure 1d (shown as *). This hints to an incomplete crystallisation reaction.

Crystallite sizes, *D*, has been estimated by using the Scherrer equation [29]:


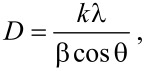


where λ is the X-ray wavelength (1.54056 Å), β is the full width at half maximum (in radian) and θ is the Bragg angle and *k* = 0.89 for composite. Crystallite size ranges between 42 to 32 nm (Table 1).

**Table 1:** Crystallite sizes of Ba_1−_*_x_*La*_x_*Co*_x_*Fe_12−_*_x_*O_19_/polyaniline composite.

sample	*x*	β (°)	*D* (nm)

COP	0	0.224	41
CL1P	0.1	0.254	36
CL5P	0.5	0.277	33
CL6P	0.6	0.218	42

### Mid-infrared spectra analysis

**Figure 2:**
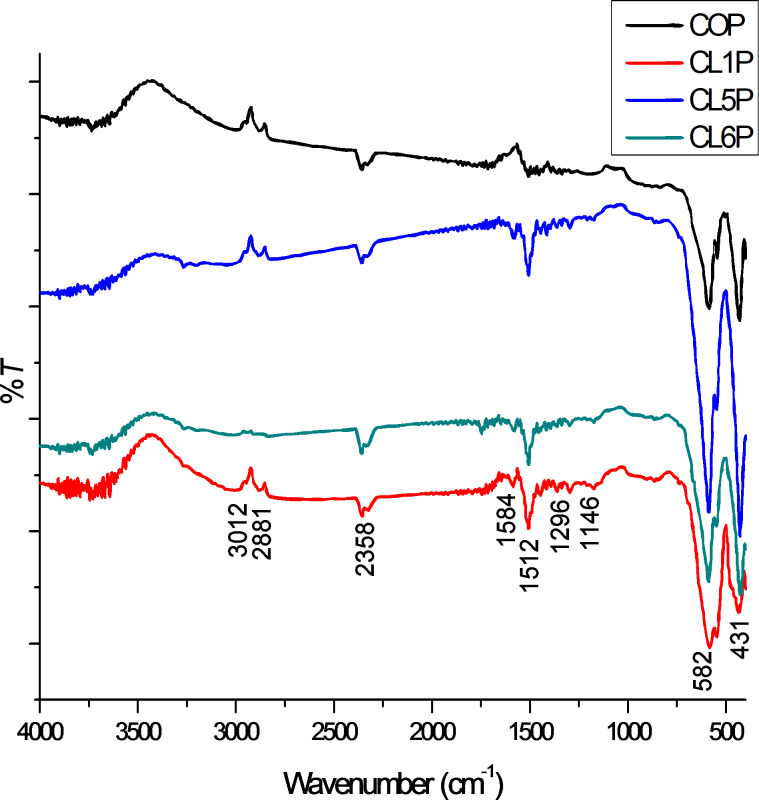
Mid-infrared region spectra for composites COP, CL1P, Cl5P and CL6P.

FTIR spectra have been recorded to identify molecular bands and functional groups (Figure 2) that are residues of the synthesis process. The relative intensities of peaks have slight variations but the peak positions remain unchanged with substitution. The formation of hexaferrites has been indicated by two prominent peaks near 430 and 580 cm^−1^. The stretching vibrations of metal–oxygen bonds (with ν_1_ and ν_2_ modes) are the reason for these peaks. The bands near 420–480 cm^−1^(ν_1_ mode) and 550–590 cm^−1^(ν_2_ mode) can be attributed to the vibration of ferric crystallographic site (octahedral or tetrahedral coordination) [30].

The broad spectral vibration in the region 1585–1146 cm^−1^ can be assigned to N–H deformation and C–N stretching. The peak at 1584 cm^−1^ is attributed to stretching vibration of C=N, the peak at 1296 cm^−1^ is assigned to stretching vibrations of the benzenoid rings. The peak at 2358 cm^−1^ is assigned to the –N≡N– diazonium salt. The peaks at 1512 cm^−1^ and range 1146–1174 cm^−1^ are attributed to in-plane deformation vibrations of C–H and in-plane C–H bending vibration mode in N=Q=N, where Q represents quinoid ring system. The band at 1146 cm^−1^ is expected due to polarization of aniline monomer. The broad absorption band near 3000 cm^−1^ is ascribed to the N–H stretching mode of the rings [31–33].

### Electron spin resonance spectroscopy

**Figure 3:**
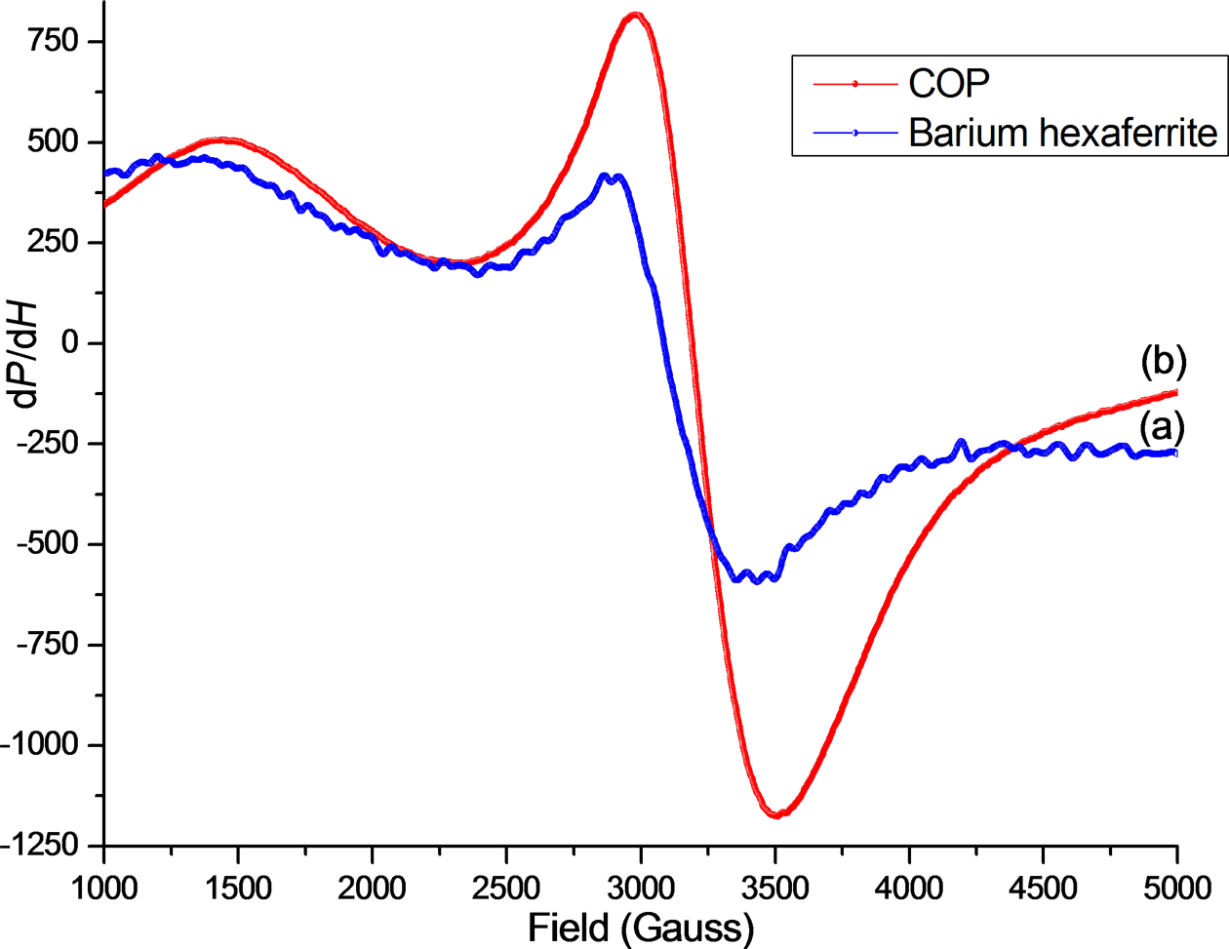
ESR spectra for (a) barium hexaferrite and (b) composite COP at room temperature in the X band.

ESR spectra of barium hexaferrite and nanocomposites were recorded at room temperature in the X band. The considerable effect (or influence) of polyaniline can be seen in Figure 3. The ESR spectra parameters (*g*, ∆*H* and *T*) have been evaluated from the Figure 3 and reported in Table 2. It can be seen that the composite COP is exhibiting a broad and intense first derivative signal. Super-exchange interaction causes anti-ferromagnetic coupling at interstitial sites of Fe^3+^ cations in BaM. These cations then show ferromagnetic resonance, which causes symmetric resonance absorption with line width ∆*H*. Two peaks that are associated with Fe^3+^ ions at tetrahedral and octahedral positions of hexaferrite grains are observed in the ESR spectra. The spectra are exhibiting two resonance parts. One can be called low-field resonance part near *g* = 4.49 that is a characteristic of tetragonally coordinated Fe^3+^ in a low field having magnetically isolated high spins (*s* = 5/2) and the second part is at a high field near *H* = 3250 G with *g* = 2.12 (Table 2), mainly contributed by the Fe^3+^ ions present at octahedral sites. It has been observed that the peak intensity is increased in the composites as compared to pure barium hexaferrite [34].

**Table 2:** ESR parameters of barium hexaferrite and COP taken at room temperature in X band.

sample	∆*H* (G)	*g*	*T* (s^−1^)

barium hexaferrite	531	2.12	5.83 × 10^−11^
COP	524	2.12	5.90 × 10^−11^
PANI [35]	1.88	2.00	1.74 × 10^−8^
PANI:BaM (1:1) [35]	34.43	1.99	9.55 × 10^−10^
PANI [36]	1.073	2.00	3.05 × 10^−8^
PANI:BaM (1:1) [36]	35	1.99	1.89 × 10^−8^

The interaction of the spins of electrons and exchange of energy with adjacent atoms is related to the relaxation time and can be calculated from value of ∆*H* by using following relation:

[1]
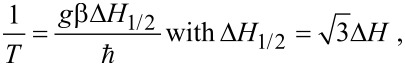


where β is the Bohr magneton (9.274 × 10^−21^ erg·G^−1^), Δ*H*_1/2_ is half of peak to peak width, and 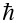 is the reduced Planck constant with a value of 1.055 × 10^−34^ J·s. The relaxation time is smaller than other values reported earlier. It means the degree of delocalization is smaller in the case of our composite. The linewidth has larger value than the values reported in [5,31,36]. The samples are showing only small changes in the line width but the intensity increases significantly. The increase of electron conductivity (measured conductivity of aniline is 0.28 mS/cm) due to presence of polyaniline and the interaction of polyaniline with hexaferrite affect the intensity of the resonance lines. The intensity is proportional to the number of spins (concentration of paramagnetic species that have a single unpaired electron) taking part in the resonance. The increase in peak intensity is only significant for a *g* value of 2.12 or near 3250 G but not near *g* = 4.49 (or *H* = 1500 G). This behaviour can be explained on the basis of the concentration of spins. The peak at *g* = 4.49 is mainly because of tetragonally coordinated Fe^3+^ ions. Thus, polyaniline has not influenced on the signal intensity. However, at *g* = 2.12, which is the approximate value of a free electron, the electrons of polyaniline are enhancing the intensity of the signal. The width and the area of the absorption peaks depend on the interaction of the present spins with their environment and on the number of unpaired spins. The obtained composite have a high spin number, which results in a strong interaction between the polymer and the magnetic material and, therefore in a broad linewidth in the ESR spectrum [35]. The interaction between the spins of hexaferrite and polyaniline are effecting the motion of π electrons, which causes an increase the absorption curve area [5,35].

### Magnetic properties

**Figure 4:**
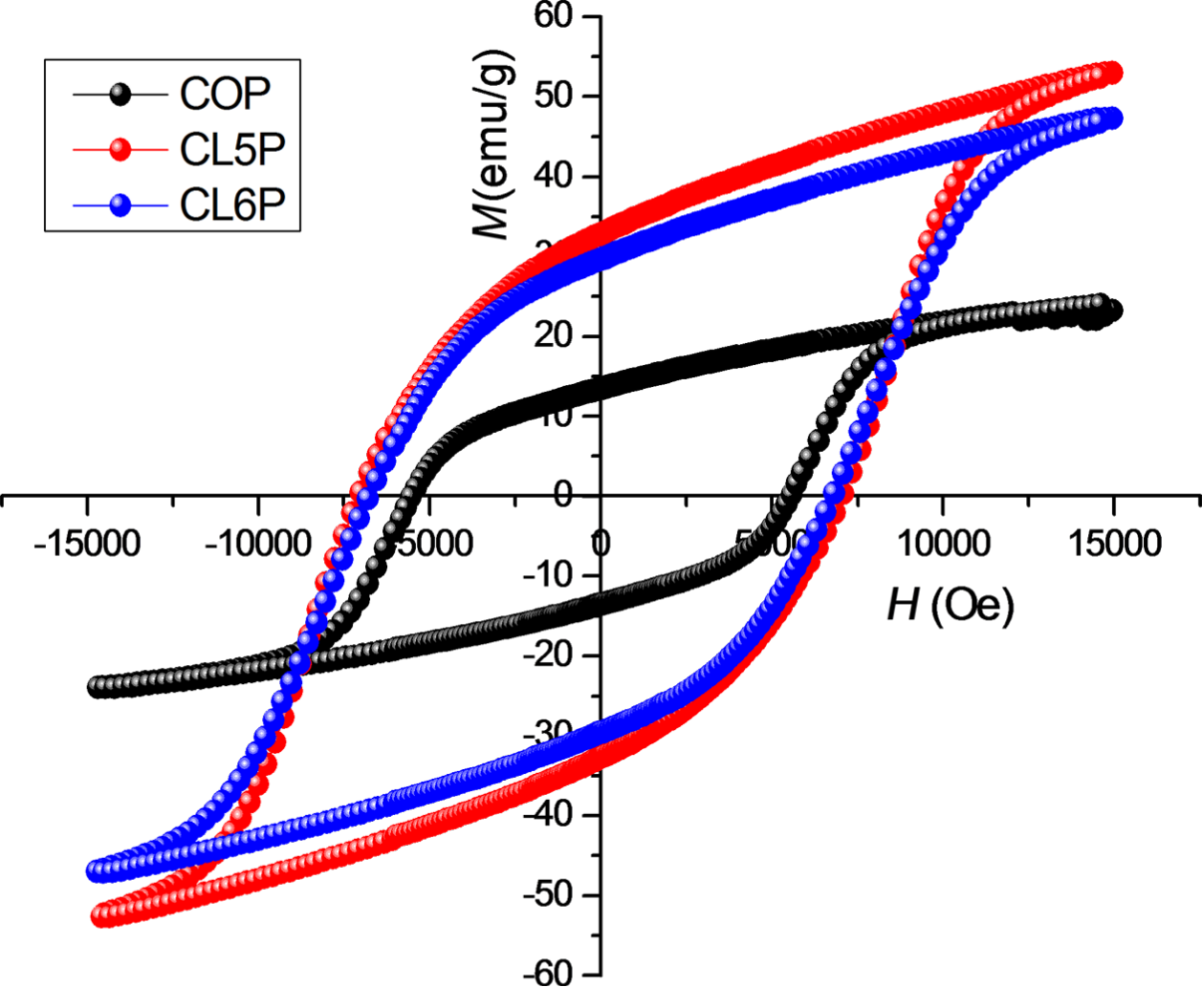
Hysteresis loops of composites.

Hysteresis loops for PANI/Barium ferrite composites recorded at room temperature are shown in Figure 4. The magnetic parameters evaluated from the *M*–*H* curves are reported in Table 3.

**Table 3:** Coercivity, saturation magnetization and remanence of PANI/barium hexaferrite.

sample	*H*_C_ (G)	*M*_S_ (emu/g)	*M*_r_ (emu/g)

COP	5560	23.61	13.30
CL5P	6774	52.65	29.54
CL6P	7096	47.06	32.71

The highest value for saturation magnetization is 52.65 emu/g at an external applied field of 15 kOe. The composites are exhibiting a smaller saturation magnetization than pure barium hexaferrite as reported elsewhere [37]. Hexaferrite is the only magnetic component. The nonmagnetic coating layer of polyaniline reduces the saturation magnetization. The substitution has increased the coercivity. The purpose of substitution was to enhance the magnetic properties to study the effect of radiation absorbance [4]. The grain size affects properties such as initial permeability, line width, domain-wall displacement and coercive force. The new absorption mechanism arises from the reduced particle size in nanometre range. The natural resonance frequency of barium hexaferrite lies in the range of 50–60 GHz because of the large magneto-crystalline anisotropy and high saturation magnetization (72 emu/g) [38]. But the weakening of magnetic anisotropy shifts the resonance to lower frequencies. The resonance frequency directly depends on saturation magnetization and coercivity [39]. The surface effects become prominent when particle sizes are in the nanometre range and their properties become different from those of the bulk material [38].

### Surface features

**Figure 5:**
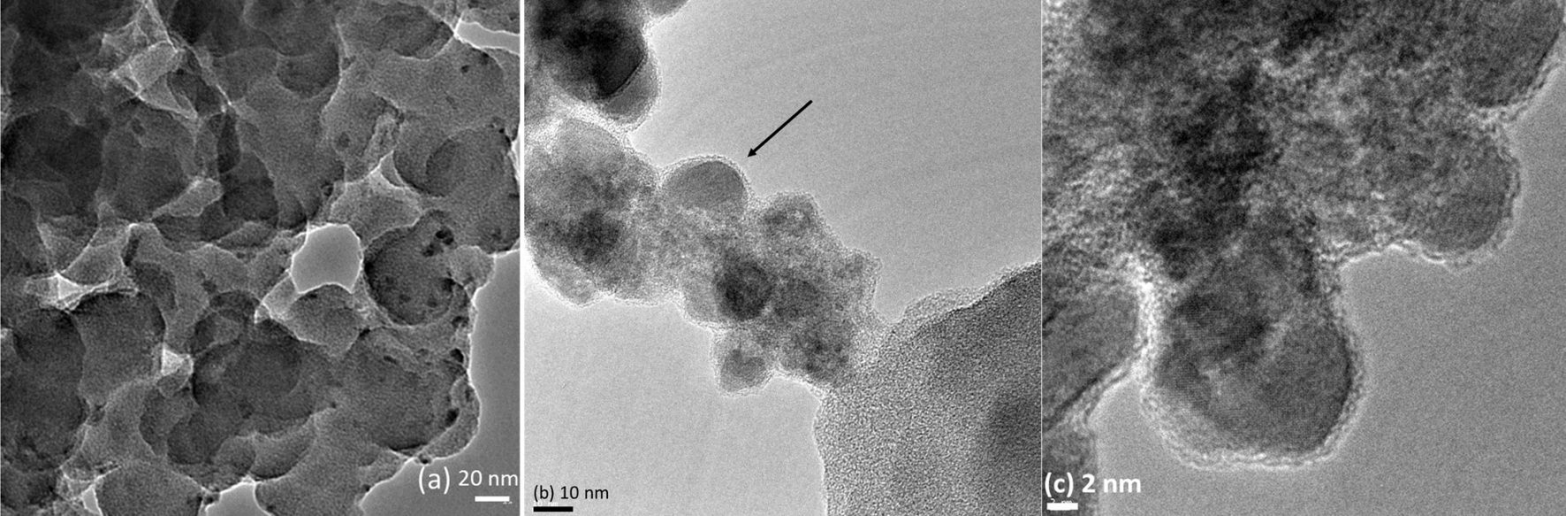
Transmission electron micrographs of magneto-electric composites (a) cluster of composites (b and c) particles of hexaferrite enclosed by polyaniline (CL6P).

Transmission electron microscopy (TEM) has been used to determine the distribution and morphology of hexaferrite nanoparticles and polymer. A clear picture of a core–shell morphology can be seen in the TEM micrographs. Figure 5 shows the hexaferrite particles coated by a continuous layer of amorphous polyaniline as shown by arrow in the image. These particles are similar to those reported by Ohlan et al. [40]. The polyaniline is unevenly distributed and the particles form clusters owing to magnetic inter-particle interaction [5].

### Radiation loss study

**Figure 6:**
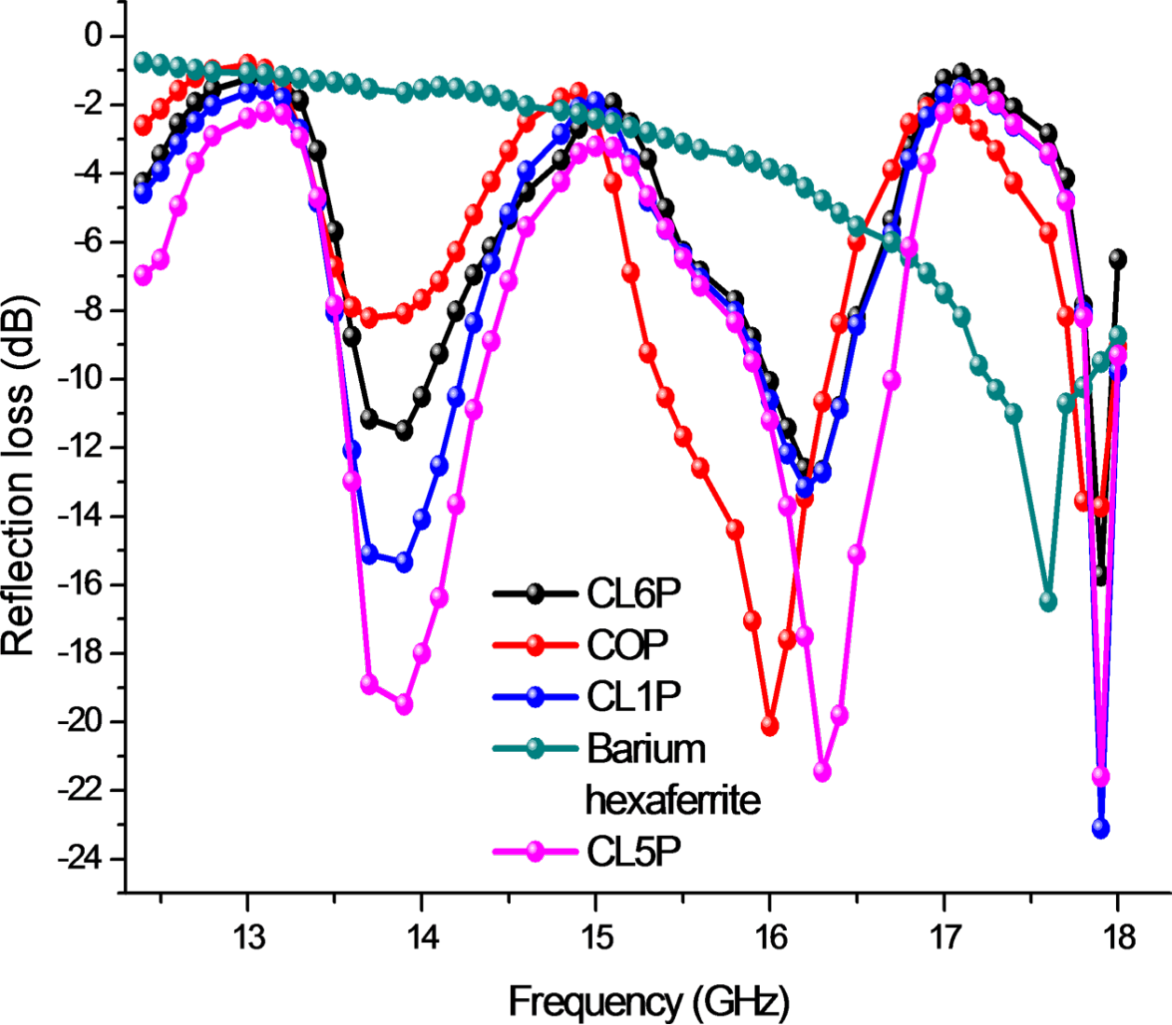
Reflection loss for composites.

The dependence of the calculated reflection loss for composite samples on the frequency in the range of 12.4–18.0 GHz (K_u_ band) is shown in Figure 6. A distinct pattern reveals that the reflection loss depends on the presence of polyaniline and the magnetic properties of hexaferrite. The width of the loss band is almost equal for all composite samples at a particular frequency but intensity varies, that is because of saturation magnetization and coercivity. Consecutive peaks are present after every 2 GHz. The reflection losses of different composite samples are CL5P: −19.45 dB at 13.89 GHz, −21.48 dB at 16.31 GHz, −21.61 dB at 17.92 GHz; CL1P: −15.32 dB at 13.86 GHz, −13.16 dB at 16.20 GHz, -23.01 dB at 17.90 GHz; CL6: −11.43 dB at 13.85 GHz, −12.59 dB at 16.20 GHz, −15.75 dB at 17.93 GHz; COP: −8.27 dB at 13.72 GHz, −20.08 dB at 16.00 GHz, −13.86 dB at 17.87 GHz; barium hexaferrite −16.53 dB at 17.59 GHz.

**Table 4:** Maximum reflection loss for composites.

sample	maximum radiation loss (dB)	frequency of maximum radiation loss (GHz)

CL5P	−21.61	17.92
CL1P	−23.01	17.90
CL6P	−15.75	17.93
COP	−13.86	17.87
barium hexaferrite	−16.53	17.59
PANI/BaM [5]	−12 (approx.)	7.9 (approx.)
PANI/BaTiO_3_/BaM [35]	−21.5 (approx.)	36 (approx.)
PANI/BaM [36]	−12.5	7.8

**Figure 7:**
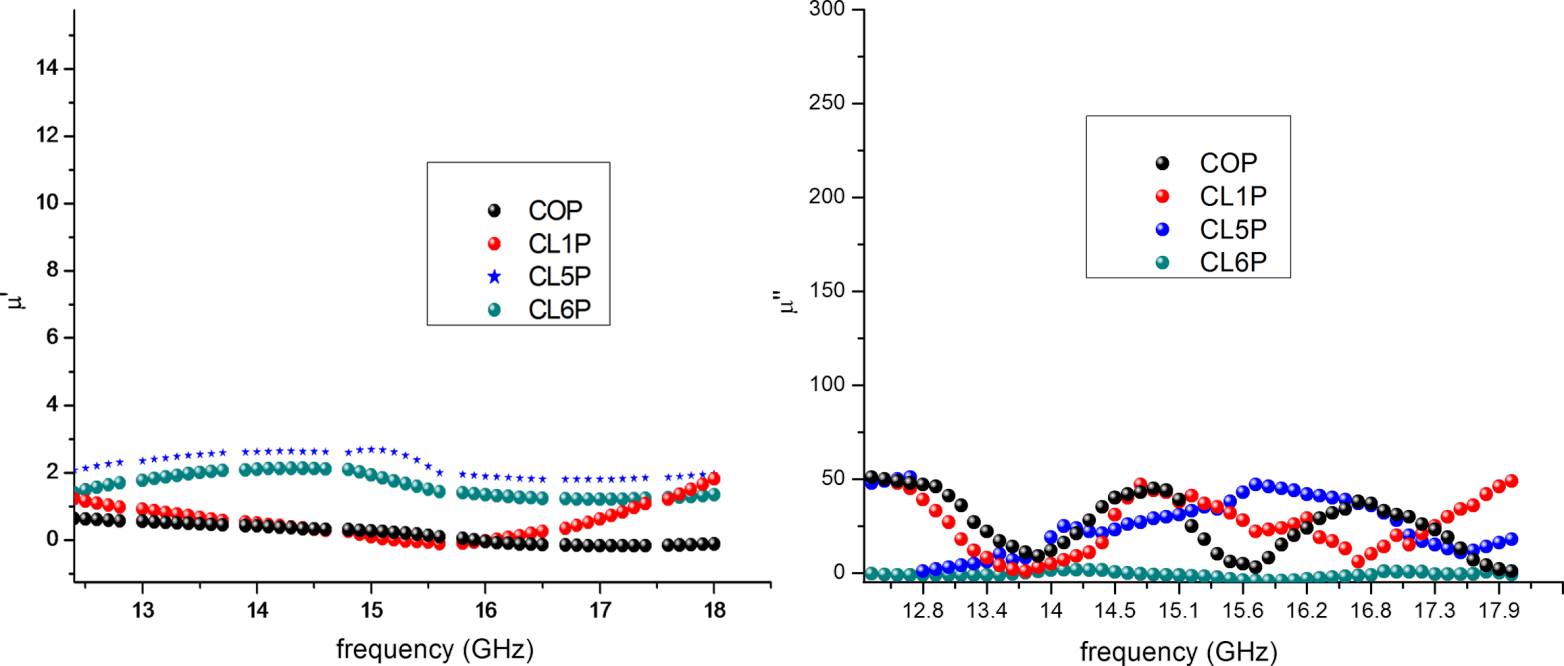
Real (µ′) and imaginary part (µ″) of the complex permeability (µ).

**Figure 8:**
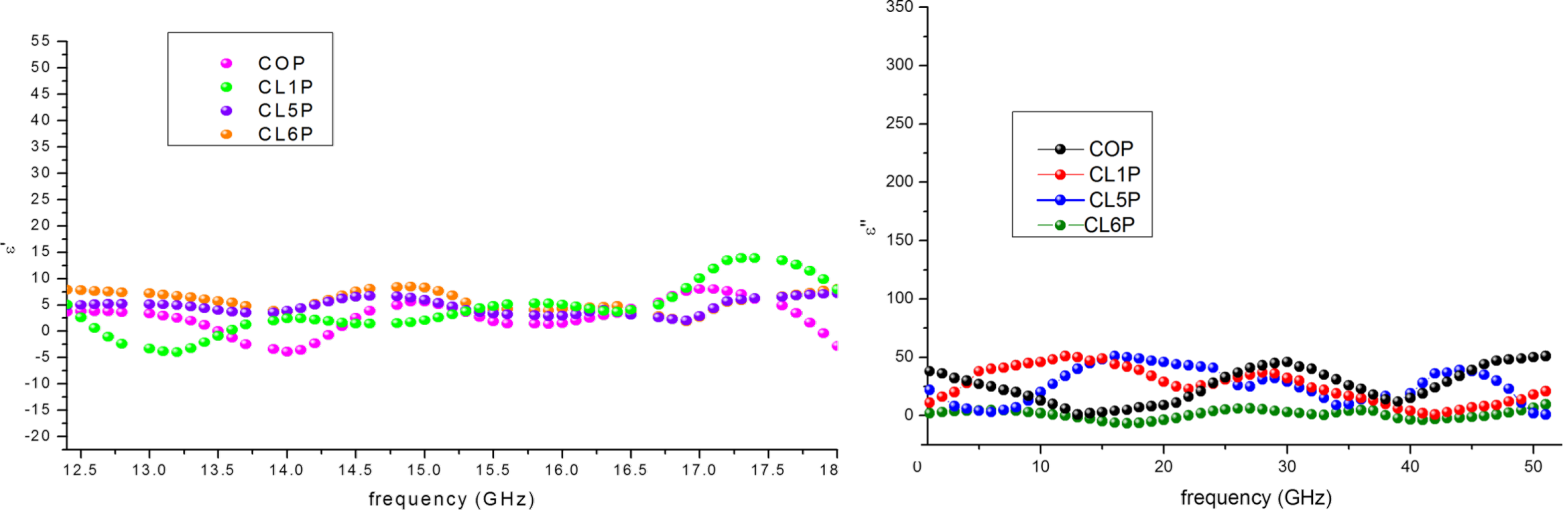
Real (ε′) and imaginary part (ε″) of the complex permeability (ε).

Sample CL5P shows better properties among the prepared composites because of the high saturation magnetization. The synthesized material can be utilized as a radar-absorbing material. The reflection loss of the composite is larger than that of hexaferrite alone. This may be due to the electrical properties of polyaniline. Multiple reflections, due to the embedding of ferrite in polyaniline and polarization, because of electron hopping between ferric ions and magnetic losses collectively, increase the reflection loss. For radiation loss measurements, samples have been moulded in rectangular shape using an aluminium die. Polyvinyl alcohol (PVA) is used as a hardener to enhance the strength of the pellet. The incident electromagnetic wave continually gets reflected and scattered within the PANI and embedded particles and get entrapped causing a loss of energy. Reflection loss calculation has been carried out by using the input impedance from the following relations in accordance with theory of absorbing wall [41]:


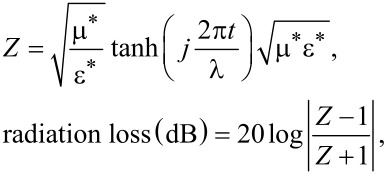


where *Z* is the normalized input impedance, ε* is complex permittivity and µ* is the complex permeability, λ is the wavelength and *t*is the thickness of the sample pellet. It has been observed from XRD and VNA analysis that reflection loss depends on size of the crystallite size. Permittivity and permeability are calculated according to Nicholson–Ross–Weir method. Figure 7 is showing the real (µ′) and the imaginary part (µ″) of the complex permeability and Figure 8 illustrates the real (ε′) and the imaginary part (ε″) of the complex permittivity of the composite. Permittivity and permeability show a variation with the frequency. A resonance in the X-band frequency occurs because of the resonant frequency of electron hopping (Fe^3+^ ↔ Fe^2+^). The imaginary part of permeability contributes more to the losses because of the occurrence of ferromagnetic resonance. The higher permeability values cause a shift of the ferromagnetic resonance frequency [42–43]. The matrix of conducting polyaniline and hexaferrite contributes to the dielectric losses (ε″) and to the dielectric constant (ε′). The unsaturated coordination on the surface, nano-sized hexaferrite, the dangling bond atoms, and the enhanced surface area lead to multiple scattering resulting in the loss of radiation.

Quantum size effects generate a separation among energy levels. Upon incidence of microwave having energy equivalent to the size of spacing of discrete energy levels, the electron can absorb and leap from one to another level causing a loss of energy. It has been observed that at lower frequencies, the band widths of loss are larger than those at higher frequencies. Moreover the losses increase at higher frequencies.

## Conclusion

The composite material has been successfully synthesized through emulsion polymerization. The XRD measurements show a crystalline structure and the formation of nanocomposites. FTIR spectra provide evidence for the presence of ferrite particles and polyaniline with low intensity peaks. Compared to pure barium hexaferrite, the coercivity of the samples increases whereas the saturation magnetization of composites seems to be far lower. It has been concluded that the magnetic properties have a considerable effect on the radiation absorption. Consecutive peaks of radiation loss of about −20 dB are present in the plots. ESR spectra show the participation of polyaniline electrons in increasing the intensity of the signal. TEM micrographs reveal the enclosure of ferrite particles in polyaniline. The prepared material shows good reflection loss values, consequently, it can be used as radar-absorbing material.
